# Social and health impacts of exoskeleton use on care workers

**DOI:** 10.1177/10519815251336912

**Published:** 2025-05-14

**Authors:** Riika Saurio, Satu Pekkarinen, Pihla Säynäjäkangas, Satu Mänttäri, Ari-Pekka Rauttola, Juha Oksa, Helinä Melkas

**Affiliations:** 1 School of Engineering Science, Lappeenranta-Lahti University of Technology LUT, Lahti, Finland; 2 Finnish Institute of Occupational Health (FIOH), Helsinki & Oulu, Finland

**Keywords:** exoskeleton, care work, social and health impacts, qualitative impact assessment, physiological measurement

## Abstract

**Background:**

In many countries, the growing shortage of care workers versus the growing number of people needing care is causing concern. In this healthcare transformation, exoskeletons, a form of wearable robotics, have been introduced as a means to aid care workers in their physically demanding and ergonomically challenging work.

**Objective:**

This study investigated the human impacts of exoskeleton use in care work. We followed a mixed-methods research approach, presenting a novel combination of qualitative and quantitative research.

**Methods:**

We conducted a three-week trial of an exoskeleton with eight assistant nurses in a round-the-clock care home. Qualitative data consist of pre-interviews, post-interviews and user diaries. Quantitative data were collected via physiological measurements.

**Results:**

Several types of social and health impacts of exoskeleton use were identified. The qualitative analysis revealed physical, mental, work practice and learning-related impacts. The exoskeleton was most useful in bed care activities. The physiological measurement results demonstrated a trend-like reduction in muscle activity throughout the workday and in different work tasks when using the exoskeleton. The exoskeleton did not have an impact on metabolic strain or perceived exertion but did increase perceived discomfort after the measured work shift when using the exoskeleton.

**Conclusions:**

Both the qualitative and quantitative results suggest that there is potential for exoskeleton use in care work environments if sufficient attention is paid to its essential prerequisites. These include the care work and tasks in question, care workers’ interests and competences, the environment, planning and organisation of the work, managerial practices and clients’ health.

## Introduction

Technology has profound impacts on all areas of modern life, including work and human resources management practices.^1^ Technology is also changing and shaping the healthcare sector, the context of this study, in a variety of ways.^2^ A need to investigate the co-existence of technology and people and the different kinds of impacts that technology has on its users has emerged (e.g.,^3^).

A variety of theoretical approaches are aimed at understanding how technologies are integrated into people's lives and daily practices. Some, such as the diffusion of innovations,^4^ mainly concern societal-level integration, whereas others, such as technology domestication theory (e.g.,^5,6^), concern the household or organisational level. In addition, there are approaches for tackling the relationship between people and technology at the individual level. Technology adoption and acceptance models represent such approaches and include the technology acceptance model^7^ – adapted from the theory of reasoned action^8^ – and the unified theory of acceptance and use of technologies.^9^

Implementation research is a somewhat more recent field that also informs decisions about healthcare services, practices and policies and their delivery.^10^ It can consider any aspect of implementation, including the factors affecting it, processes and results (such as “implementation outcomes”;^11^), to understand what, why and how solutions and interventions work in “real-world” settings and how they could be improved.^10,12^ These research fields and approaches may provide novel perspectives to management, as managerial practices are important in helping employees adapt to new workplace technologies (e.g.,^13^).

In this article, we follow a mixed-methods approach, recommended as a practical way to understand multiple perspectives and types of outcomes,^10^ and present a compilation of qualitative and quantitative research on exoskeleton implementation, care workers and care work, focusing on human impacts of the use of the device in a broad sense. This perspective can be regarded as complementing existing technology diffusion, domestication and acceptance models, as well as implementation research traditions. According to recent studies, there is a lack of knowledge related to the benefits of and obstacles to technology use, which affect technology acceptance.^14^

In Finland, the context of this study, as well as in many other countries, concerns have arisen about the growing shortage of care workers versus the growing number of people requiring care.^15^ During this period of change, exoskeletons, a form of wearable robotics, have been introduced as a means of assisting care workers by reducing their physical exertion.^16^ Exoskeletons have also been used with good results in other fields, such as manufacturing.^17^ More than a third (37%) of the work tasks of assistant nurses are physically demanding, which is connected to sick leaves due to musculoskeletal disorders (6.5% of working time).^18^ One of the most exertion-inducing care tasks is the transfer of clients in poor physical condition in round-the-clock housing services,^19^ which this study focuses on.

Active and passive exoskeletons have been developed to support different parts of the body.^17^ According to ASTM International,^20^ an exoskeleton is “a wearable device that augments, enables, assists, and/or enhances physical activity through mechanical interaction with the body.” An exoskeleton may include either rigid or soft components, or both, according to the same standard. Electric motors or other actuators are deployed in active exoskeletons to increase the wearer's strength and to reinforce the wearer's joints. In passive exoskeletons, materials such as springs are deployed to store energy from human movement and then use it to support the wearer's posture or movement.

Research concerning the benefits of exoskeleton use is still scant. Apart from a few exceptions, studies have focused on fields other than care services.^16,21,22^ These studies have indicated the beneficial effect of an exoskeleton in reducing back loading in simulated work settings. Technical development has generally been examined (e.g.,^23^) instead of physiological exertion or ease of use. Research on implementation, ease and comfort of use^24^ and impacts on workers and work communities has been called for.^25^ Benefits, work ergonomics, ease and comfort of use and attitudes affect willingness to use the devices.^16^ Few studies have focused on the field of care, and qualitative and quantitative approaches have not been combined. Only a few studies have included physiological exertion measurements in real-world care work.

Research is needed to clarify exoskeletons’ suitability for care work and how they should be implemented. Exoskeletons could potentially extend care workers’ careers and increase the number of future care professionals. However, implementing technology in care services is demanding and complicated; therefore, it is necessary to think carefully about how to introduce new technological tools and what exactly is needed.^16,26^ In this study, both qualitative impacts on users and the results of physiological measurements are presented. This is accomplished with a field study concerning the impacts on users of the Auxivo LiftSuit 2.0 exoskeleton (hereafter also referred to as “suit” or “device”) in the care of older adults, conducted in a care home in Finland. The human impacts of implementing this new device as a tool at work are examined by looking into individual-level experience-based and physiological impacts, as well as (wider) social impacts. The research question is: What are the human impacts of the use of exoskeletons in care work? It is further divided according to social and health impacts, as follows:

– How is exoskeleton use experienced by care workers in their daily work (e.g., what opportunities and limitations are associated with exoskeleton use)? (Social and health impacts)
– What are the measured impacts on physical exertion in care work? (Health impacts)

Furthermore, we discuss managerial implications, such as how to strengthen the positive impacts and mitigate the negative impacts identified in the study.

## Exoskeletons in care work

Technology plays a significant role in care service renewal, especially in addressing the sustainability gap in the services for older adults.^27^ High hopes are placed on technological innovations such as e-health solutions and home automation, safety monitoring, robots, and simpler applications.^28^ At the societal level, Finland has adopted governmental strategies to promote the technological development of public services (https://vm.fi/en/digitalisation), including care services.

However, balancing economic sustainability with social sustainability is not an easy task, and various confrontations may appear when a balance is sought between a human orientation and increased efficiency.^29^ Technology in the care sector is still often depicted as a “separate island” with poor connections to its contexts.^30^ To benefit from technology use, functional and operational rethinking are needed, which are time-consuming processes.^14^

The introduction of exoskeletons for the benefit of care workers and their physically demanding and ergonomically challenging work has the potential to meet the high hopes concerning technology use. Work-related musculoskeletal disorders are among the most common health problems in Finland and are the main cause of sick leaves. In 2022, musculoskeletal problems were the most common reason for outpatient doctor visits and disability pensions, and 41% of Finnish women surveyed experienced back pain in the prior month.^31^

Back-exerting work, especially frequent heavy lifting, awkward working postures and vibration, are connected to the prevalence of back problems. The shape and weight of the load, as well as its position in relation to the body at the beginning and end of the lift, affect the loading of the back. Repeated lifting causes cumulative load on the back and further increases the risk of overloading. Sudden and uncontrolled movements during lifting, such as when the grip is suddenly released, can damage muscles, joints or tendons.^32^ Measures related to work ergonomics are one way to prevent musculoskeletal disorders in the workplace.

In addition to traditional work ergonomics, exoskeletons can be used to assist with lifting. Exoskeleton manufacturers recommend their devices as effective aids for reducing physical workloads, but little scientific research evidence has been gathered from realistic work situations. According to subjective assessments, they have positive impacts, but due to their weight, for example, they can also negatively affect the physical load. Exoskeletons have been studied in various tasks in laboratory environments. According to recent systematic reviews,^24,25,33^ an exoskeleton that supports lower back muscles and facilitates lifting reduces the load on the lower back muscles in both static and dynamic tasks. Lower back muscle activity has been found to decrease significantly, by up to 75%. Back-support exoskeletons also reduce perceived exertion, compressive loading of the spine and the metabolic demands of lifting work. However, exoskeletons may also have adverse impacts, such as experienced discomfort^34^ and increased muscle activity in other regions, such as the abdominal or lower limb muscles.^24^

These exerting postures occur frequently in care work, even if work ergonomics are taken into consideration. Coping and staying in care work are challenges for working life and society as a whole, and concerns about workforce retention have increased even more during the COVID-19 pandemic.^35^ The use of exoskeletons in care work has barely been studied, and studies have focused on short tests.^22,26,36^

## Methods

### The exoskeleton

**Figure 1.**
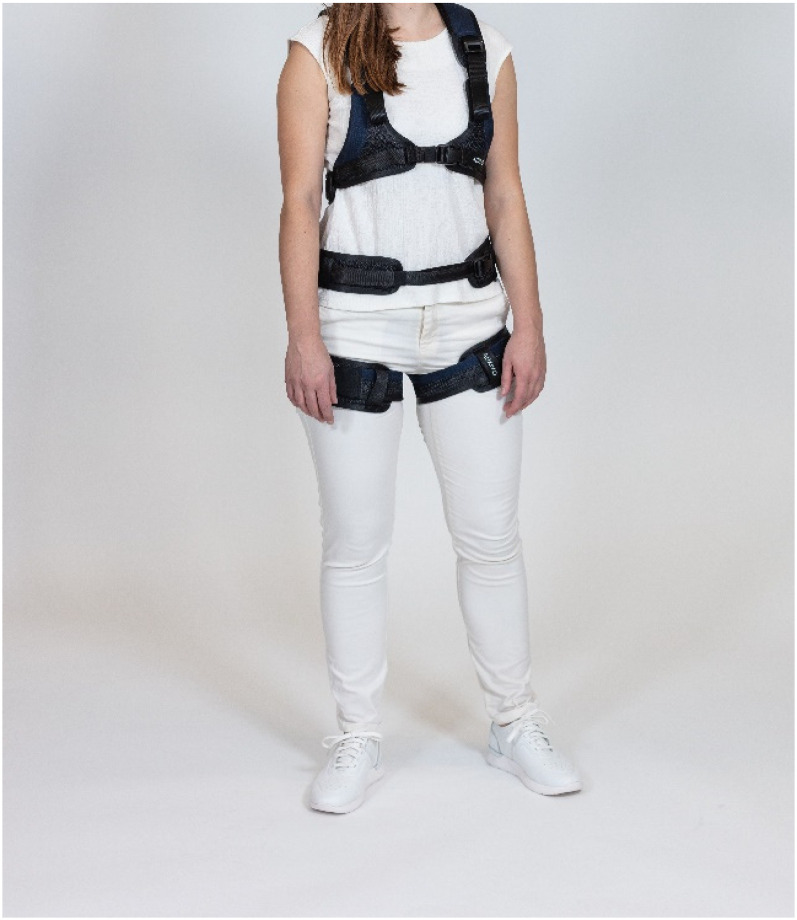
Liftsuit from front (Source: Auxivo AG).

**Figure 2.**
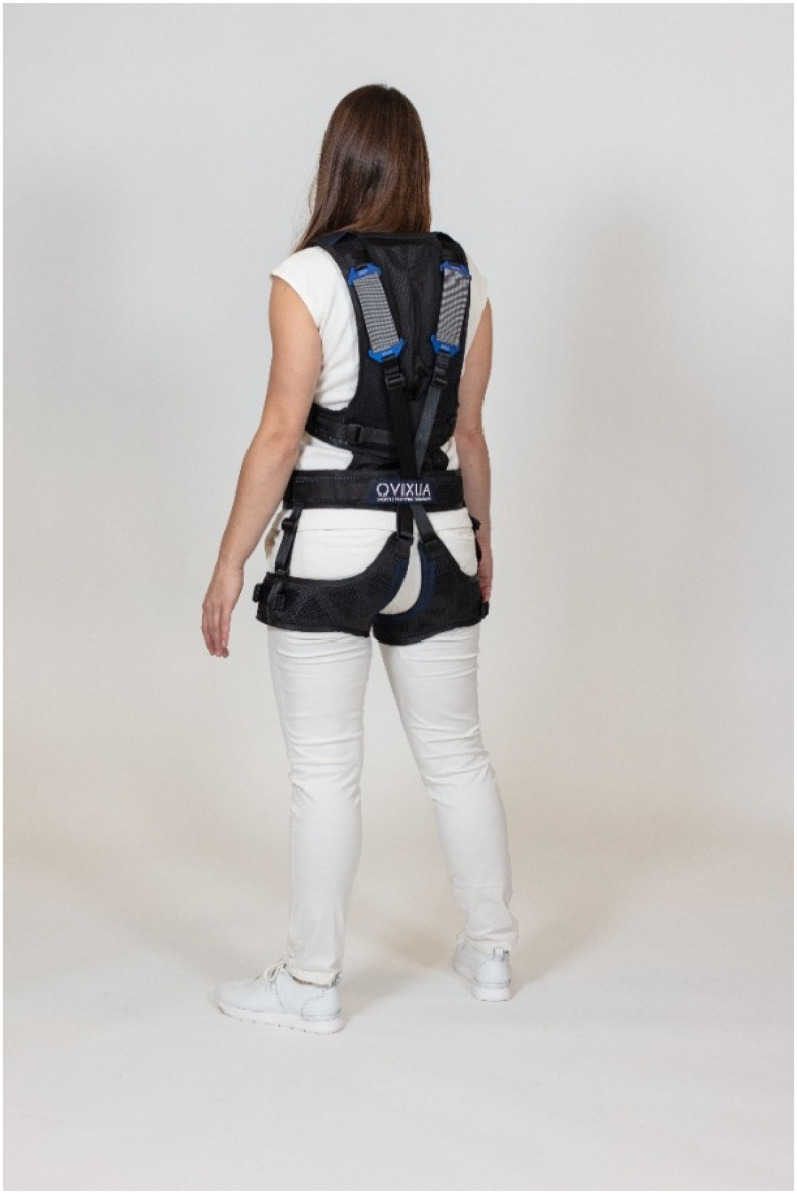
Liftsuit from behind (source: Auxivo AG).

This study focuses on the Auxivo LiftSuit^®^ (Figure 1 and Figure 2), a passive Swiss-made exoskeleton. It supports the back when the user lifts objects below waist level or works in a forward-leaning position. Elastic textile springs on the back store energy that, when released, supports the wearer's movements. The support is activated and deactivated by straps in the front. The suit is available in two basic sizes and is designed to accommodate wearers of different sizes through an adjustment mechanism. The suit weights 0.9 kg in size S/M and 1 kg in size L/XL and it can be machine-washed at 60°C and wiped with disinfectants. Safety loops, which are rubber bands that can be attached to the straps of the adjustment mechanisms to prevent them from hanging, help ensure safety during use.^37–39^

### Research environment, participants and data collection

The research environment was the Pohjola care home, located in and operated by the city of Tampere, Finland. Pohjola provides 24-h housing services for older adults. Eight assistant nurses (seven women and one man) were recruited on a voluntary basis for the qualitative part of the field study. Seven took part in the quantitative measurements (one was absent due to illness). Their age range was 19–61 years, and their care work experience ranged from 1.5 to 40 years. The participants were divided into two groups. Each participant was able to use an Auxivo LiftSuit exoskeleton for three weeks (four assistant nurses at a time).

Qualitative data were collected through pre-interviews, post-interviews and user diaries written during the study (see also^38,39^). Quantitative data were collected through physiological measurements (^38^; see also^40^). This research was part of the TUEKS project “Exoskeletons and Caregivers’ Changing Daily Work”, supported by the Finnish Work Environment Fund. The number of participants was considered adequate for this type of research (see^16^). Assistant nurses were chosen as there are no other care workers in fieldwork in care homes in Finland.

Prior to the pre-interviews, on the same day, a comprehensive orientation and introduction to using the exoskeleton was given to the participants by a researcher with a nursing background. The introduction used a slide show format to describe the purpose of the suit and possible barriers to use. Instructions for putting on the exoskeleton were presented in a video. Each participant received individual assistance in putting on and taking off the suit. The participants then lifted a chair in a lifting exercise with and without activating the device. This exercise demonstrated the support that could be expected from the device. Finally, the participants received an instruction manual in their native language and the telephone number of the researcher to call if they had any problems. The post-interviews were conducted when the devices were collected right after the three-week testing period.^38^

The interview questions covered expectations for the use of the exoskeleton, its experienced impacts on the user and their work, needs regarding introduction and orientation, and reactions of others to the use of the device. Information on immediate experiences and the times and purposes of use was collected in user diaries. The participants were instructed to fill in the user diaries throughout the testing, making observations on use, especially ‘at the extremes’ (if/when the device was not working or was working well). The user diary also served as a support for the post-interview, which was informed to the participants. On the other hand, it was emphasised that they should not feel pressure about filling in information on every moment in their busy work. The structure of the user diary was as follows: user name, date (of a specific observation) and columns for the following questions on the observation:

1. For what purpose and for how long did you use the device?
2. How was the experience: How did you feel about using the device? What worked well? What was difficult in the use?
3. Comments from clients, their relatives and colleagues? Any other observations?

The interviews were recorded and transcribed. Ethical approval for this research was obtained from the Regional Ethics Committee of the Expert Responsibility Area of Tampere University Hospital, Finland (approval number R22026). The research was conducted according to ethical standards to avoid any participant harm. The care workers gave their informed consent to participate in the study. Their safety was ensured, the research material was anonymised and no personal information could be identified from the data.^38,39^

Physiological exertion measurements were conducted in authentic work environments, including during typical nursing duties (e.g., bedside care, food supply, toileting assistance, bathing the patient, lifting and transferring the patient). Participants were measured during two work shifts: one without the exoskeleton (REF) and one with it (EXO). During the measured work shifts, the participants performed their work tasks as usual. Electromyography (EMG), heart rate (HR) and metabolic equivalent (MET) were measured throughout the work shift. Measured EMG activity reflects the loading on the musculoskeletal system, while HR and MET indicate the cardiovascular strain associated with the work tasks performed. Ratings of perceived exertion (RPE) and perceived discomfort were assessed before and after both work shifts.^38^

EMG was recorded with an ME6000 biomonitor (Bittium Wireless Ltd, Finland) from eight muscles: *m. deltoideus pars acromialis* (medial deltoid), *m. trapezius pars descendens* (upper trapezius, right and left), *m. trapezius pars transversa* (upper back)*, m. longissimus dorsi* (lower back, right and left sides), *m. biceps femoris* (back of the thigh) and *m. quadriceps femoris* (front of the thigh)*.* All measurements were made on the right side of the body, except for the upper trapezius and lower back, which were measured on both sides. Single-use surface electrodes (BlueSensor M-00-S, Ambu, Denmark) were placed on shaved, alcohol-cleaned skin. EMG signals were recorded at 1000 Hz, amplified (×2000), filtered with Butterworth filter (40/500 Hz) and averaged over 100 ms time windows. EMG data were recorded and analysed using MegaWin software (Mega Electronics, Finland). Before the first work shift, voluntary maximal isometric contractions were performed for each muscle. The maximal amplitude value (μV) was used to calculate the %MEMG value (percentage in relation to maximal EMG activity) for each measured muscle as well as an average for all muscles.^40^ To ensure attachment and to avoid detachment of the electrodes during the work shift they were firmly taped on the skin with adhesive kinesiology tape (RockTape, Durham, UK). Taping also helped in reducing the risk for motion artefacts during work. If motion artefacts occurred, they were removed during the analysis phase.

HR was measured with a Firstbeat Bodyguard 2 device (Firstbeat Technologies Ltd, Finland). The device was attached to the subject's chest with two single-use electrocardiogram (ECG) electrodes (BlueSensor VL-00-S, Ambu, Denmark): one electrode was attached under the clavicle on the right and the other on the rib cage on the left. MET was calculated using Firstbeat Lifestyle Assessment software (Firstbeat Technologies) based on HR and HR variability measurements.^40,41^

RPE was assessed using the Borg 6–20 RPE scale;^42^ on this scale, 6 corresponds to “no exertion” and 20 corresponds to “maximal exertion”. Perceived discomfort was assessed with an 11-point visual analogue scale; zero corresponds to “comfortable” and 10 corresponds to “uncomfortable”. In both cases, each participant was asked to choose one number that corresponded to the perceived exertion and discomfort of their entire body at that moment.^40^

### Data analysis

The qualitative data (the interviews and the user diaries) were analysed using qualitative content analysis and data categorisation.^43^ Such analysis emphasises an integrated view of speech or texts and their specific contexts and, going beyond merely counting words or extracting objective content from texts, examines meanings, themes, and patterns that may be either manifest or latent in the text in question.^43^ The analysis comprised inductive and deductive phases. After transcription, the interview and user diary data were reduced to essential content and inductively coded using an interpretive approach that involved searching for and naming recurrent themes. The Human Impact Assessment (HuIA) approach was used to identify the diverse impacts of using the exoskeleton on the participants. It offers a concrete, user-oriented approach to analysing various nuances of technology use from the individual and social perspectives.^44^ The HuIA aims to uncover the different impacts on people without following a predetermined framework and provides information for making decisions and resolving possible conflicts.^45^ It has been used to assess the impacts of “traditional” older adult care technologies, such as safety alarms (e.g.,^30^), and emerging technologies, such as care robots.^44,46^ Its essence is the holistic identification of positive, negative and neutral impacts on different groups of people. Confrontations between technologies and practices, for example due to the hard pace of work, various fears, lack of sense of participation or lack of introduction^29^ surface with the help of HuIA. Conducting HuIA regularly at the individual and workplace levels may facilitate technology adoption by users.^30^ The approach takes into account that impacts can be planned or unintended and can result from long chains or networks of impacts.^46^

The origin of HuIA is in integrating social (SIA) and health (HIA) impact assessments.^45^ In this study, we re-emphasise this original integration in our combination of qualitative and quantitative research concerning social and health impacts. Multi-perspective and multi-method impact assessments – and especially combinations of qualitative and quantitative methods in research on the impacts of the use of emerging technologies in the healthcare sector – have been called for recently, as such impacts may be multidirectional and intertwined. Qualitative assessment provides in-depth knowledge related to people's thinking and ways of doing things, which can be improved or reacted to with development measures (e.g., training) at the workplace level. In addition, qualitative understanding helps in the interpretation of quantitative assessments.^46^

The physiological measurement data were analysed using IBM SPSS Statistics 27 (IBM, USA). The data were divided into five work tasks based on work diaries filled out by the employees during the measurements. The work tasks were (i) bedside care, (ii) food supply, (iii) assisting with toileting, (iv) lifts, transfers and making the bed and (v) bathing the patient. The data were tested for normal distribution using the Kolmogorov–Smirnov test. All physiological data followed a normal distribution. Differences in %MEMG, HR and MET between REF and EXO were compared with the paired samples t-test. The level of statistical significance was set at *p *< 0.05. All results are presented as mean ± standard error of mean (SE).^38^

## Results: social and health impacts of exoskeleton use

By analysing the qualitative data as well as physiological measurement data, we identified several types of social and health impacts of exoskeletons. They were categorised as described in Table 1 and are examined in this section. As the interview and user diary data were combined for the analysis, the results are integrated.

**Table 1.** Categorisation of results concerning social and health impacts of exoskeleton use.

Qualitative impacts – social and health impacts	Quantitative impacts – health impacts
Experienced physical impacts	Muscular strain
Experienced mental impacts	Metabolic strain
Impacts on care work practices and processes	Perceived exertion and discomfort
Impacts on learning needs	

### Qualitative results – social and health impacts

Four types of social and health impacts on care workers were identified in the qualitative analysis. They have negative, positive and neutral dimensions and are described below with quotations from the interviews.

#### Experienced physical impacts

Regarding negative experiences, some participants felt that there was “something extra” on their bodies that restricted movement, especially when bending down to, for example, assist with putting on adult diapers, pants and socks. Extra weight and stiffness were considered disturbing. The device was felt to clutch the chest and thighs, and it was not possible to adjust it sufficiently for diverse body types. One participant over 50 years of age felt that the suit was a bit burdensome and distressing to wear, making it more appropriate for younger care workers. The participant felt that as the skin and body become less flexible with age, the suit becomes uncomfortable.

The participants mentioned that the suit restricted work tasks other than repetitive work. If the user does not have well-developed thigh muscles and has, for example, varicose veins, rheumatism or osteoarthritis, use may not be possible. One participant experienced sudden hip and knee pain at the beginning of use, but not later.

“I have an illness, so those activation straps are really bad. I suffered pain. I can’t pull them. I have rheumatism, that's it. Should be a different system. It doesn’t work for me.”

Assisting during toileting and other tasks, such as helping residents with their shoes, done in a squatting position were especially challenging, as the exoskeleton felt clumsy. It was more difficult to get up with the tight and compressing suit.

“When I had to squat, it was a bit irritating that it was so compressing. It felt useless in those situations.”

The participants also brought up the constant need to adjust the straps. Despite that they sometimes got tangled in the edges of the bed.

During showering tasks, it felt sweaty because an apron is needed, and it is otherwise hot in the room. This is difficult, especially for people with sensitive and atopic skin.

**Figure 3.**
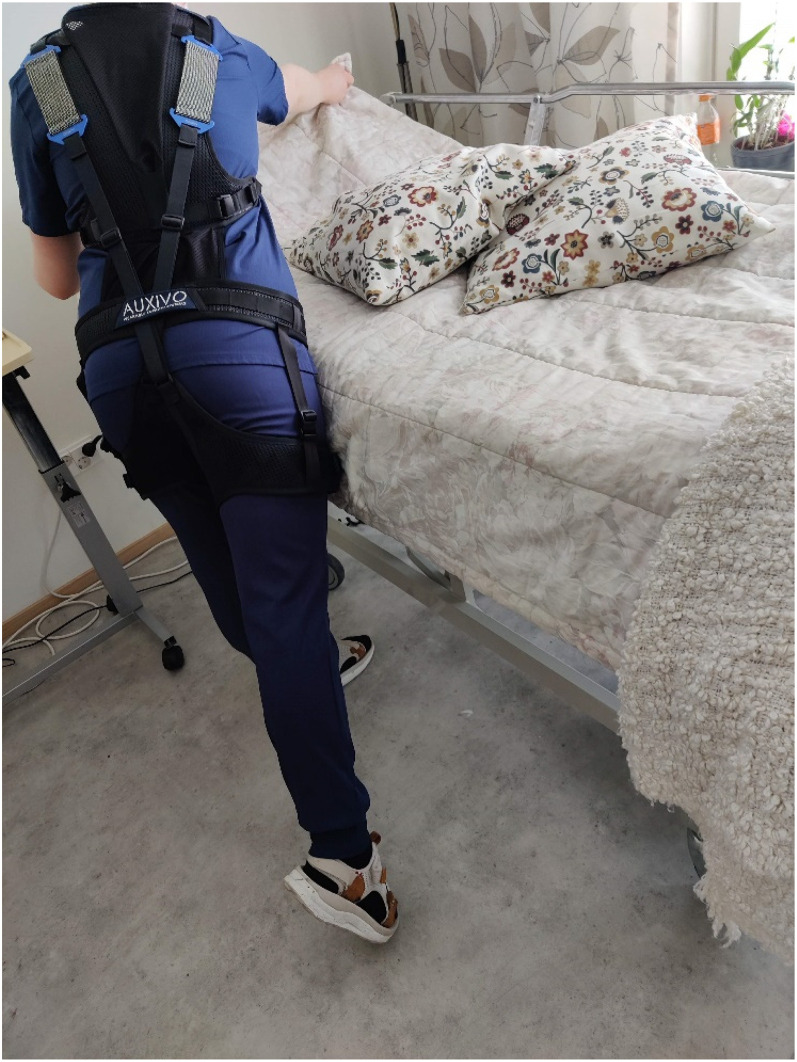
Straightening the bed covers (Source: Riika Saurio, 2022).

As for positive experiences, the participants mentioned that they had been more cognisant of ergonomics and had been more careful in physically demanding situations, which may have made it more challenging to discern the suit's benefits (Figure 3). The added professional value of using the exoskeleton was seen to consist of a better understanding of one's own body and how to use it at work. The suit was felt to keep the posture better and make the user automatically straighten up, which is good as the participants mentioned that they tend to stoop.

“Maybe I thought a bit more about the posture […] in daily work. More about ergonomics. Even though I like to raise the beds to a good height and so […] maybe I thought even more about how I pull this and do that… That was the benefit. And it was nice to try something new.”

**Figure 4.**
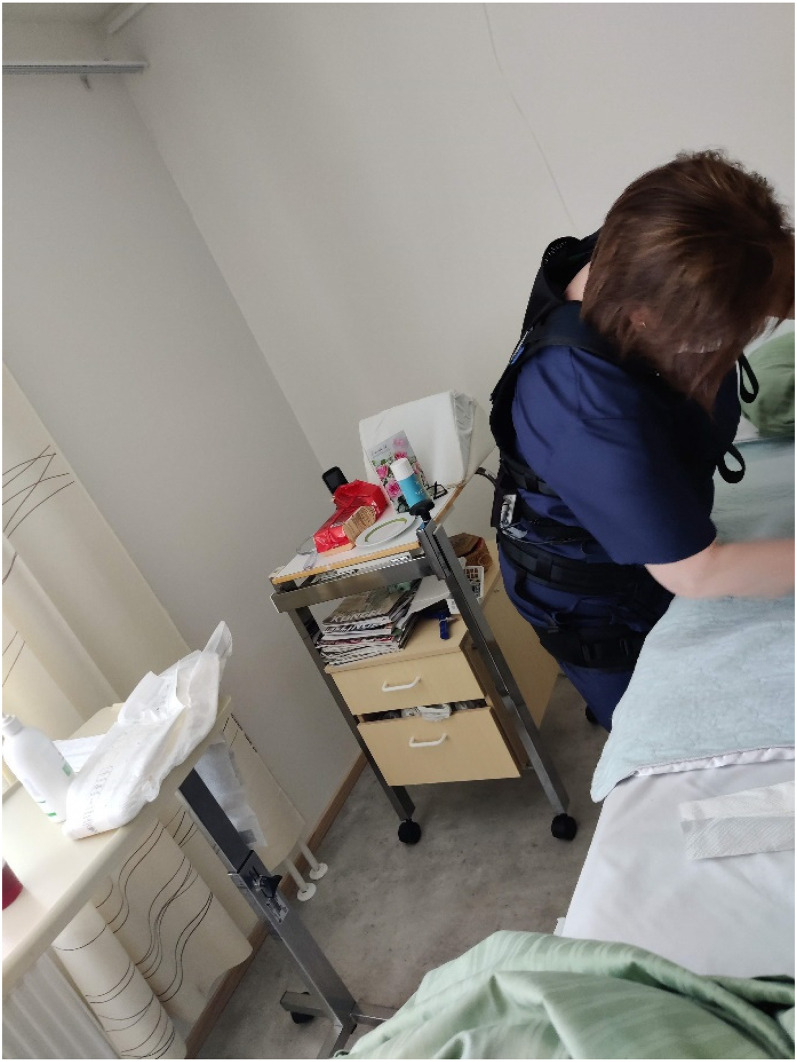
Bedside morning care (Source: Riika Saurio, 2022).

The suit was found to be most appropriate for bed care (Figure 4), such as bed washing, posture shifting and changing diapers, because it supported the lower back. It was also useful for assisting the resident from the bed to the showering platform and for showering in the sauna facilities. Assisting residents in wheelchairs to the toilet and from there to the bed was easier with the suit. During feeding and mealtimes, it helped with body rotation.

The device was also useful in reaching positions. For example, one participant experienced this during bed washes, where they sometimes had to reach out and bend down over the bed depending on the body part they were washing. In those situations, it was found positive that the device provided support for their back. One participant also mentioned that existing back pain did not worsen during use, and after the trial, their back no longer hurt.

Once the suit had been pre-fitted, it was generally found to be easy to put on and take off. Sometimes it was difficult to find the right strap and there were challenges in putting on the thigh parts. The plastic parts were somewhat slippery. Two participants expressed a desire for a rougher material or for ball- or T-shaped activating straps. The suit itself was thus found to be easy to use, but there were issues with comfort.

#### Experienced mental impacts

Regarding negative experiences, exoskeleton use did not become routine and resulted in increased cognitive load, which the nurses felt added to their hectic workload. The biggest challenge was remembering to activate and deactivate the suit. A few times the user forgot to activate the suit during a task and performed the task without it. Overcoming this barrier will likely require reminders and getting used to the exoskeleton.

During the trial, some participants also felt that they thought too much about the feedback they should provide for the research and that they were not as present with the residents as they normally were. However, this is related to the research context and not directly to the suit or its use.

Regarding positive experiences, the participants found it convenient that the suit was unobtrusive. One participant mentioned that it is just one assistive device among others assisting work, not much to think about. The blue suit did not stand out from the nurses’ blue clothing. As a result, the residents and their relatives did not pay much attention to it. When they did, it was positive attention; they asked questions and started discussions about the suit. A few participants found it disturbing that some residents paid attention to the suit.

Some residents at the care home had cognitive disabilities, and the participants wondered if that was the reason for the lack of response to the suit. However, most of the residents’ family members also did not pay much attention to the suit, although some asked about it. The residents were positive about the suit. Some of them were a little worried but the participants saw that as general worrying that is shown as compassion towards the nurses.

The participants were consciously aware that they were using the suit. They had specifically reconsidered their work tasks and ergonomics and thought they were privileged to be able to participate in the testing.

“I personally feel very privileged to have been able to test something like this new thing, and it's been really cool to test it […] there's a bit of good and then a bit of bad […] there's a lot to develop…”

Some participants noted that there is significant societal value in raising awareness of nurses’ need for wellbeing and support in their physically difficult work, and in making technological developments such as exoskeletons known and used. This is a way to give voice to nurses, which is important in comparison to other service areas and in relation to clients. The participants emphasised the need to develop solutions and work practices that enable better coping at work in an ageing society and the nurses’ hope – “a societal call” – for better recognition and appreciation of care work. One participant mentioned that, fortunately, they haven’t had to see nurses carrying beds without wheels anymore, which is staggering, and emphasised the evolution of ergonomics over the last few decades.

In terms of neutral experiences, the suit was typically considered a normal or traditional work aid and had no specific connotations. Interestingly, however, some participants referred to it using the traditional Finnish male names Esko and Eki.

The participants shared their experiences of the suit with each other and discussed its benefits and disadvantages. The majority felt that the discussion was neutral, but one participant mentioned that it had been negative. The participants had given practical tips to each other, for example, related to the adjustments. Some older workers discussed chafing, wondering if the suit would be better for younger workers with more solid bodies. Understanding how the suit fits different bodies at different ages is essential.

How exoskeleton implementation is communicated within the organisation affects mental impacts and its potential for long-term use. The communication should be “soft” and have a participatory rather than imperative tone. The participants emphasised that early communication was key, and urgency should be avoided. In the care sector, technologies are typically forced on workers by management, and imperative, urgent messaging often leads to resistance.

“Care workers are pretty good at looking after themselves and understand the importance of support and help. This is hard work, but it is not good that we always have to go through the compulsion [of using new technology].”

#### Impacts on care work practices and processes

Regarding negative experiences, having more to remember at work – activation and deactivation – increases the already high workload. If the device is not assigned and adjusted to one nurse, there is a concern that its use may end. Nurses may believe that tasks can be done more quickly without the device, because time spent fetching and putting on the exoskeleton could be spent on their care work instead.

“Better organising would probably promote the use […]. If you just could get it into use right when you need it […]. There are troubles in using it […]. You should put it on, and if you don’t get the benefit from it, then it just makes things go slower. You then must remember extra things.”

The starting point for the field study was that the suit could be used as much as possible in all work tasks. Therefore, its use did not require extra planning or changing the order of tasks. This was felt to be positive. The participants didn’t really plan the use, they just wore the device and “let's see…”.

However, it was noted during the study that regular use of the device requires proper planning. Being able to reserve the suit for certain tasks, such as showering, was hoped for.

A neutral finding was that the visibility and availability of the device in the workplace was considered necessary to ensure that the nurses remember to use it. The participants felt that storing the exoskeletons in lockers, for instance, would make them less convenient to use. The suits need to be visible so that the nurses in general remember that they exist and can be used.

For the device to remain in use in daily work, the provision of a proper introduction and orientation was emphasised. A feeling of mastering the use of the device is a necessity, and the related skills must be mastered before adjusting the suit to one's own use becomes easy.

The participants saw hygiene – how the suit would be washed or disinfected – as a practical challenge that requires planning as another prerequisite for use. The suit is washable, but could it be washed with residents’ clothing or other laundry, or should it be washed separately? Could it be used by several nurses before it needs to be washed? When is disinfection sufficient? It was suggested that if the device is assigned to one nurse, it would be washed once a week and disinfected daily, several times if necessary. When it is visibly dirty, machine washing is always necessary.

#### Impacts on learning needs

Impacts on learning needs were also identified. They were neutral, very practical and intertwined with the other types of impacts.

Mainly, the introduction and orientation were felt to be clear and useful, and there were enough and different kinds of learning materials. Some had challenges getting a good feel for the suit and making the right adjustments. Remembering to activate and deactivate the suit was the biggest challenge. Sometimes, work was already done before remembering, or the activated suit was worn unnecessarily. The participants stated that a longer trial period would probably promote learning and discerning the positive impacts; only the challenges may be visible at first.

Very practical information was appreciated most during the orientation. People need to understand what the exoskeleton is all about, how it is used and why. Individual side-by-side guidance during actual work tasks was hoped for. The participants emphasised that the trainer should always understand the type of work that the nurses are doing. This should be taken as the starting point for the introduction and orientation. The participants praised the instructor's own nursing background “as a nice addition” and felt it showed that the instructor had a good perspective on the use of the suit.

The participants stated that they would have liked to have had a hands-on demonstration in real working conditions with residents. The instructor could then have told them when to activate and deactivate the suit. Information about situations in which the participants could benefit most from the suit, such as changing diapers when the resident is unable to leave their bed, would have been important. One participant mentioned that they don’t lift chairs at work as they did during the introduction, and that trying the exoskeleton in practice would give them a sense of learning on their own, which could lead to better internalisation of use.

The three-week trial period was mentioned to be too short for the exoskeleton to be truly integrated into care practice. In three-shift work, some nurses ended up having few shifts during which they could use the suit. The testing period should last at least six weeks, or even two to three months. This would make the suit a more natural part of the work, according to the participants. People's diverse characteristics affect how they learn and adopt new things, such as using the exoskeleton or other technology, which should also be taken into account.

Managers may end up with new responsibilities, such as guidance and monitoring. The participants mentioned that managers play an important role in clarifying where and how the nurses should use the new equipment, and who is involved – are the devices for everyone or only for some nurses. The managers should also motivate the nurses in a positive way, so that they actually use the devices that have been purchased and they are “not left lolling somewhere”. In addition, the managers should monitor the use, according to the participants.

Communicatively, there should probably be positive ‘marketing’ and speaking up for the exoskeleton […] by focusing on content matters, by promoting the use through work ability, coping at work, motivating … that could be [good]. In this field of care work, quite often everything seems to be forced.

All participants were willing to learn to use an exoskeleton and looked forward to further development of the device, potentially leading to increasingly positive impacts.

There was interest in the suit within the work community. The other care workers were curious about the benefits and user experiences – does the suit help and what does it feel like? Care workers from other wards and workplaces were also interested in the exoskeleton.

Overall, the discussions reflected social acceptance and were characterised by neutral attitudes. Management and the organisation were supportive of the research because they had been involved in it from the beginning. This is also felt by the participants.

### Physiological measurement results – health impacts

#### Muscular strain

**Figure 5.**
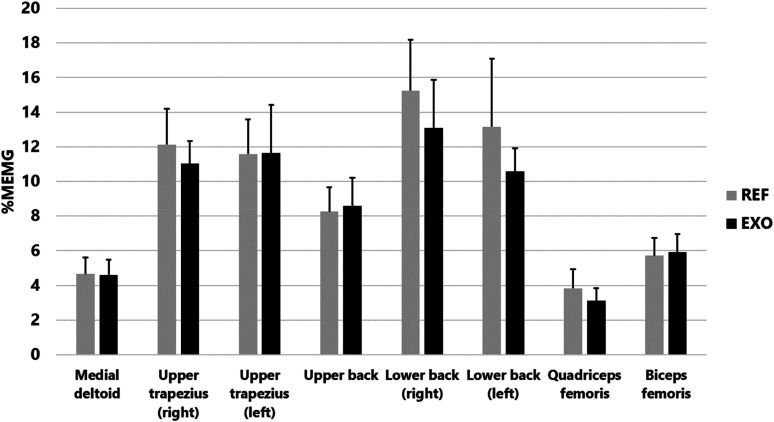
Electromyographic activity in relation to maximum (%MEMG) in eight muscles during work without (REF) and with (EXO) the exoskeleton.

Average EMG activity as a percentage of maximum activity in eight muscles during work with and without the exoskeleton can be seen in Figure 5. When looking at the entire workday, the average EMG activity from eight muscles was 9.3 ± 1.6%MEMG without (REF) and 8.6 ± 1.4%MEMG with (EXO) the exoskeleton. However, there were no statistically significant differences in muscular strain between REF and EXO in any of the measured muscles.

**Figure 6.**
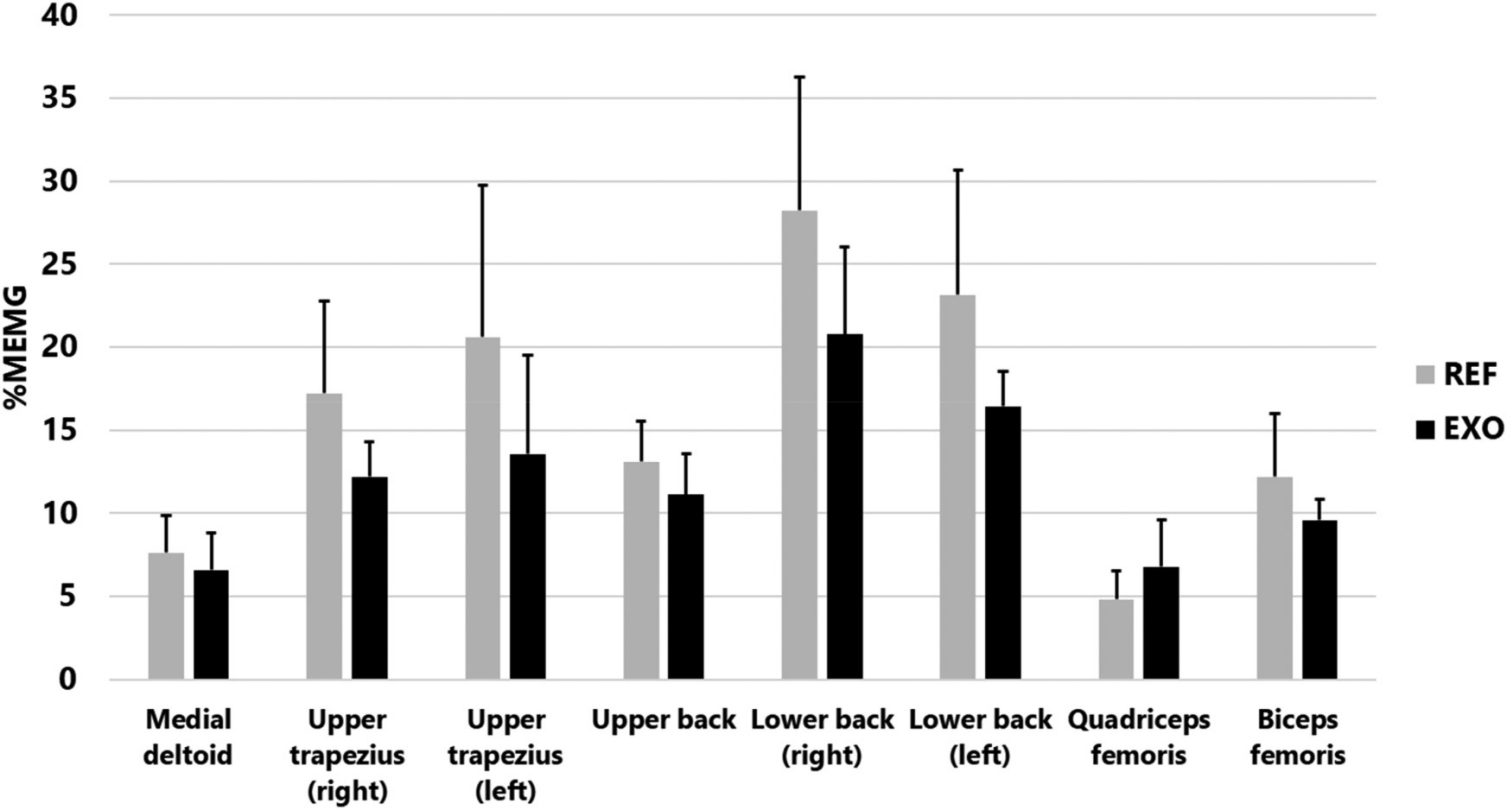
Electromyographic activity in relation to maximum (%MEMG) in eight muscles during the bedside care work task without (REF) and with (EXO) the exoskeleton.

The same trend of lower muscular strain with EXO was observed in five separate work tasks (Table 2). An example of EMG activity in REF and EXO on the bedside care task can be seen in Figure 6. However, there were no statistically significant differences between REF and EXO in the separate work tasks. Muscular strain was greatest in the lower back for all work tasks.

**Table 2.** Electromyographic activity in relation to maximum (%MEMG) as an average (± SE) from eight muscles during five nursing tasks without (REF) and with (EXO) the exoskeleton; the p-value represents the statistical difference between conditions.

%MEMG	n	REF	EXO	*p*-value
Bedside care	4	15.9 ± 3.6	12.1 ± 2.0	0.149
Food supply	4	10.8 ± 1.4	8.4 ± 1.7	0.161
Assisting with toileting	2	9.3 ± 1.3	10.4 ± 3.6	0.861
Lifts, transfers and making the bed	2	12.9 ± 5.1	10.1 ± 3.0	0.424
Bathing the patient	1	14.0	11.0	

#### Metabolic strain

**Figure 7.**
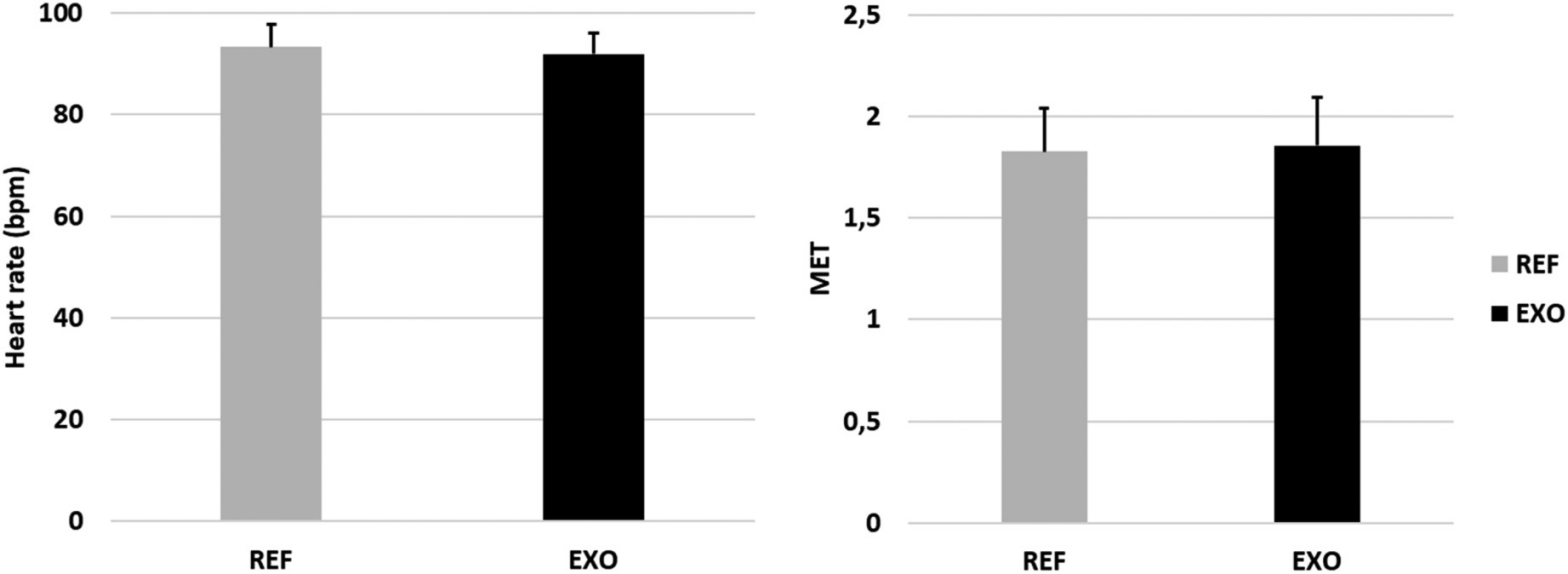
Heart rate and metabolic equivalent (MET) in care work without (REF) and with (EXO) the exoskeleton.

Metabolic strain (i.e., HR and MET) are presented in Figure 7. The average metabolic strain in care work was 1.8 ± 0.2 MET. There were no significant differences in HR or MET between the REF and EXO conditions.

#### Perceived exertion and discomfort

**Figure 8.**
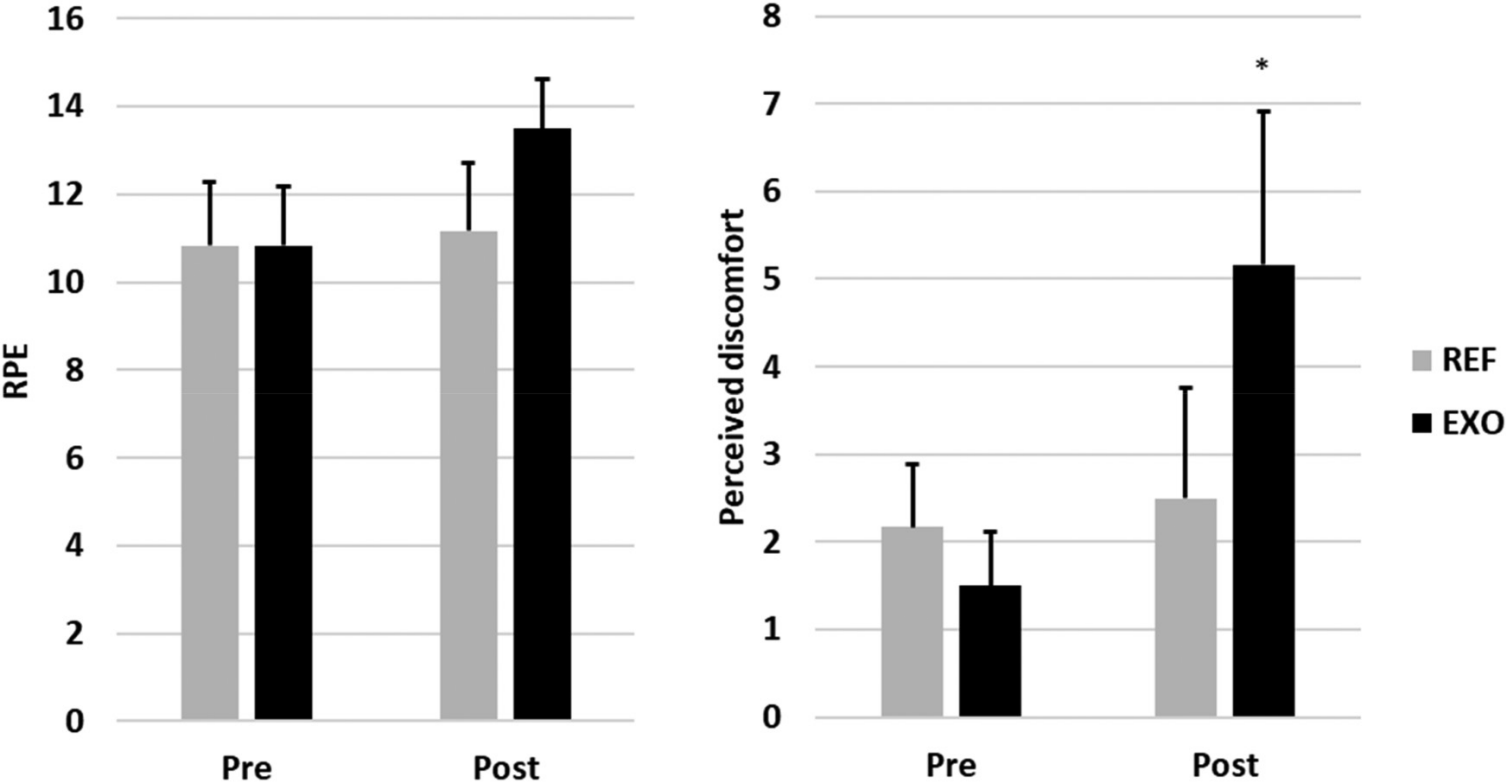
Ratings of perceived exertion (RPE) and discomfort assessed before (pre) and after (post) care work without (REF) and with (EXO) the exoskeleton. *Statistically significant difference between Pre and Post: p < 0.05.

Perceived exertion and discomfort are presented in Figure 8. RPE was similar before work in both conditions but slightly increased during the work shift in EXO. However, this increase in RPE was not statistically significant. Perceived discomfort was slightly lower before work in EXO but increased significantly during the work shift in EXO compared to the rating measured before work (*p *= 0.022). Perceived discomfort remained at the same level in REF even after the work shift. There were no significant differences between the conditions in the change in RPE or perceived discomfort during the work shift.

## Discussion

This study combines qualitative and quantitative research. The qualitative analysis revealed several types of impacts with positive, negative and neutral dimensions and showed their intertwined and multidirectional character, such as the connection between the suit's integration into work practices and mental well-being at work. Overall, there is potential for the implementation of exoskeletons in the care sector, where work tasks are often physically demanding and can easily lead to sick leave.^18^ The results of this study are encouraging in this regard, as participants identified many benefits and relatively few barriers.

The suit was felt to be appropriate especially for tasks that required leaning forward. Squatting positions caused the suit to feel clumsy and uncomfortable, which is consistent with previous studies that also reported experienced discomfort.^34^ More knowledge is needed about the work and workplace specific situations in which the exoskeleton is beneficial. Positive factors included the ease of use after the initial adjustment of the suit and its lightness and unobtrusiveness. The suit did not attract particular attention from the residents. It was seen as a neutral tool that was generally socially accepted. The study by Schreer et al.^47^ reveals that social contextual factors, such as concerns about co-workers’ or managers’ reactions (perceptions of the abilities of an exoskeleton user) as well as patient confidence on the professional abilities of the user (when receiving care from a nurse wearing an exoskeleton), are important to exoskeleton use among nurses and nurse managers in long-term care, and in this light, the perceived neutrality of the exoskeleton is an interesting finding.

In terms of practical recommendations, the qualitative research highlighted the following aspects. When introducing the suit to care workers, the benefits of using it should be clearly described. Lucid guidelines regarding the tasks for which the suit is most appropriate are critical, and a hands-on exercise related to daily tasks should be included in the introduction and orientation. The nurses need learning time and reminders to remember to activate the suit before performing appropriate tasks. The sequence of work tasks should be carefully planned. Importantly, all participants were ready to use some type of exoskeleton at work if they benefited from its use. They felt that the development and use of exoskeletons could increase societal appreciation of care work, which could lead to nurses’ voices becoming more prominent in society. However, the three-week trial period was considered too short. The need for longer trial periods should be taken into account in future research.

The quantitative research, i.e., the physiological measurements, revealed the following. When using the exoskeleton, the physiological measurement results demonstrated a trend-like reduction in muscle activity during the entire workday and in different work tasks. On average, the reduction was 7%. The greatest reductions, 20%, 14% and 18%, were observed in the right and left lower back and front thigh, respectively. When comparing tasks, the exoskeleton reduced muscle activity the most during bedside care. Such work consists primarily of forward-leaning postures, for which the Auxivo LiftSuit exoskeleton was designed. Overall, these results indicated a trend-like beneficial impact of the exoskeleton in reducing the risk of muscular overloading and musculoskeletal disorders.

Differences in HR, MET and RPE may have been expected due to the weight of the exoskeleton (0.9 kg) because the literature reports that each additional kilogram may increase metabolic strain by 1%–3% depending on the location, shape and attachment of the additional load.^48,49^ The lack of increase in cardiovascular strain can be explained by the reduction in muscle strain, which counterbalanced the impact of the extra load. However, a disadvantage associated with exoskeleton use was observed in the physiological measurements. Perceived discomfort was reported to increase significantly while using the exoskeleton. Since the other parameters did not indicate negative impacts, exoskeleton use can still be recommended from a physiological point of view.

**Figure 9.**
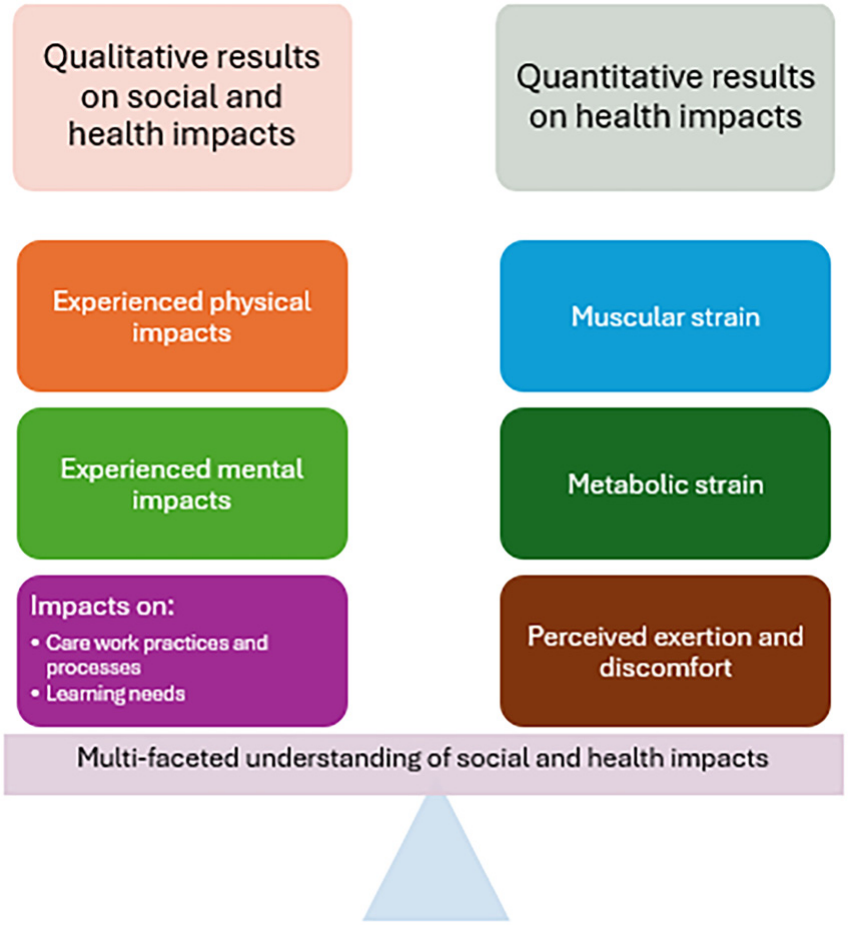
Towards a holistic and balanced understanding of different types of impacts.

Together, these results provide a multi-faceted picture of the impacts of exoskeleton use, as the qualitative and quantitative assessments complement each other (see Figure 9). This study provides real-life confirmation of previous studies of exoskeleton use conducted in simulated work settings that reported the beneficial impact of an exoskeleton in reducing back loading.^16,21,22^ The physiological measurements supported the results of the qualitative assessment – for example, the care workers mentioned in the interviews that they had felt that the exoskeleton was helpful in bedside care and the measurements supported this. The new perspective that can be developed by combining quantitative and qualitative methods makes it possible to move towards an increasingly balanced understanding of different types of impacts and to consider their optimisation (Figure 9; see also^46^) when appropriate.

Qualitative results also provide in-depth knowledge related to people's thinking and ways of doing things, which can be reacted to (e.g., with training) and help when designing managerial actions in the workplace and the organisation. Management has a crucial role in supporting exoskeleton use in care work, promoting the positive impacts and reducing the negative ones. The need for managers’ developmental support and coaching^13^ as well as the need for supportive organisational cultures and open communication channels^47^ were confirmed, including the organisation of proper orientation and participatory communication early in the process. Ensuring that the device is used for tasks for which it is most beneficial and planning the necessary changes in work processes are important managerial questions, as are concrete details such as washing practices and finding appropriate places for storage.

The number of participants and a single exoskeleton model can be considered as a limitation of this study. However, the purpose of the quantitative measurements was to support the qualitative assessment. The physiological impacts of exoskeletons have been extensively studied in laboratory settings, and the physiological measurements in this study showed a similar trend in these specific work tasks. Exoskeletons are a rapidly evolving product sector. At the time of the study design, there were only a few exoskeletons available on the market for care work applications. According to previous research,^16^ exoskeletons with metallic components were perceived as cumbersome for care work. Therefore, this study aimed to test a new model that was available at the time of the research and met the hygiene and safety requirements of care work.

The short testing period can also be seen as a limitation. Previous studies have used even shorter trial periods. In the study by Turja et al.,^16^ care workers emphasised the need for a testing period longer than one week, specifically citing an entire shift schedule cycle (three weeks) as an appropriate duration. Based on this, a similar timeframe was adopted for the present study. During the study, the participants were not under continuous observation; they were allowed to use the exoskeleton as part of their regular work routine. However, during the physiological measurements, the researcher observed two participants for approximately 30 min while they performed their tasks and took illustrative photographs. This approach was designed to minimise the influence of observation on the participants’ thoughts and behaviours. In addition, the participants completed the diaries at their own discretion, based on their personal interest.

According to this study, exoskeletons have the potential to manage and enhance the abilities and performance of employees, attract workers and motivate and retain employees, all of which are central managerial goals.^1^ More effective technology utilisation is also a significant challenge for health and care policies, which highlights the need for additional multi-method research. The exoskeleton utilised in this study is a relatively simple device compared to the active exoskeletons deployed in rehabilitation. Nonetheless, various social and health impacts were found in this study. The central prerequisites for exoskeleton use depend on several factors, such as the type of exoskeleton, the characteristics of the care work and its environment, the care workers’ interests and competences, the planning and organisation of the work, the managerial practices and the health of the clients. The findings provide guidance for care work organisations and professions ranging from grassroots care workers to development and management and exoskeleton developers. These findings pave the way for the identification of best practices for exoskeleton use – specifically the Auxivo LiftSuit – in care work, and thus for the increasingly effective technology use in future care services.
